# Non-Uniform Fusion Tree Generation in a Dynamic Multi-Sensor System

**DOI:** 10.3390/s17051020

**Published:** 2017-05-04

**Authors:** Kyuoke Yeun, Daeyoung Kim

**Affiliations:** 1 Agency for Defense Development, Yuseong-gu Soonam-dong, Daejeon 34186, Korea; 2 School of Computing, Korea Advanced Institute of Science and Technology (KAIST), 291 Daehak-ro Yuseong-gu, Daejeon 34141, Korea; kimd@kaist.ac.kr

**Keywords:** air surveillance system, track fusion, multi-sensor tracking, two-tier fusion process, fusion tree, distributed information processing

## Abstract

This paper addresses the proposal that the number of processed air tracks of a two-tier fusion process can be increased by applying a balanced fusion tree which can balance tracks across local fusion nodes. Every fusion cycle, a fusion process combines duplicate tracks from multiple radars and creates a single integrated air picture (SIAP). The two-tier fusion process divides the fusion process into local and global. The results of the local fusion process, executed at local fusion nodes, are used in the global fusion process. This hierarchical structure can be modeled as a fusion tree: each radar, local fusion node, and the central server is a leaf, internode, and the root, respectively. This paper presents a non-uniform fusion tree generation (NU-FTG) algorithm based on clustering approach. In the NU-FTG, radars with higher scores get more chances to become local fusion nodes. The score of a radar is in proportion to the number of tracks of the radar and its neighbors. All radars execute the NU-FTG independently with the information of their neighbors. Any prior information, such as the appropriate number of local fusion nodes, predefined tree structure, or position of radars, is not required. The NU-FTG is evaluated in the OPNET (Optimized Network Engineering Tool), network simulator. Simulation results show that the NU-FTG performs better than existing clustering methods.

## 1. Introduction

A fusion process is required to obtain a single integrated air picture (SIAP) with air tracks from a dynamic multi-radar system (DMRS). The DMRS consists of numerous mobile radars that are connected via a wireless ad hoc network. Air tracks from multi-radar should be fused to create a SIAP. The SIAP is the product of fused, near-real-time and real-time data from multiple radars to allow the development of common, continuous, and unambiguous tracks of all airborne objects in the surveillance area [1]. A fusion process, which factors out duplicate information [2] and improves on the state estimation of tracks with duplicate information [3], is required to obtain a SIAP for the DMRS.

The centralized fusion architecture is theoretically optimal [3], but this architecture requires high network bandwidth and high computing resources at the central server. In this architecture, radars send all observed measurements to the central server for every observation cycle. The central server executes the tracking process to create tracks and the fusion process to create the SIAP by combining tracks that originate from the same target. On the other hand, in the hierarchical fusion architecture, presented in References [4,5,6], radars execute the tracking process and the central server executes the fusion process. Every fusion cycle, radars send the created tracks to the central server to be fused. In this approach, the required network bandwidth can be dramatically reduced. The fusion cycle is much longer than the observation cycle; however, the fusion process is still concentrated at the central server. Both centralized and hierarchical fusion architectures are not suitable for the DMRS, which consists of numerous nodes.

Therefore, a two-tier hierarchical fusion architecture, presented in References [7,8], is applied for the fusion process of the DMRS. In this architecture, the fusion process is divided into local and global to reduce the number of tracks processed at the central server. This architecture consists of radars, local fusion nodes, and a central server. Radars form clusters, and one of the radars in each cluster becomes the local fusion node. This structure can be modeled as a tree which is called a fusion tree. Each radar, local fusion node, and the central server is a leaf node, internode, and the root node of the fusion tree, respectively. Local fusion nodes execute the local fusion process to create the local SIAP, and the central server executes the global fusion process to create the global SIAP with the tracks from local fusion nodes. As a result, duplicate tracks in each cluster are combined at local fusion nodes, and the number of tracks to be processed at the central server can be reduced. The two-tier hierarchical fusion architecture requires a balanced fusion tree, which balances tracks across local fusion nodes, to prevent some of the local fusion nodes from becoming bottleneck nodes. However, it is hard to generate a balanced fusion tree on the DMRS, since the possible number of fusion trees exponentially increases as the number of radars increases. Therefore, topology control, which selects the role of the nodes and the flow of the fusion process, is required to generate a balanced fusion tree.

There are numerous research works in the field of multi-sensor data fusion. However, most of the research works in this field do not consider topology control to create a balanced fusion tree. In Reference [9], various problems about multi-sensor data fusion, such as data imperfection, outliers and spurious data, conflicting data, data modality, data correlation/association, etc., is presented. However, topology control is not included. In References [10,11,12], various techniques are presented on assigning and scheduling fusion tasks to sensors. However, their application is not the correct fusion process to obtain a SIAP. In Reference [13], a multi-target tracking problem is presented, but this research work deals with the sensor deployment problems. In Reference [14], the flexible fusion structure is presented, but it considers only some adjustment on the allocation of system resources to cope with the failure of nodes. In Reference [15], the dynamic assigning of fusion roles to the nodes of a sensor network is presented. However, Reference [15] requires a task graph, which defines the number of fusion and sensor nodes, as an input. In References [7,8], the two-tier hierarchical fusion architecture is presented. However, these research works concern only fusion processes on a predefined tree structure.

On the other hand, clustering sensor nodes is an effective topology control approach [16]. There are various clustering methods in the field of wireless sensor networks (WSN), but most of the clustering methods are designed to prolong the network lifetime with the residual energy of nodes. In References [17,18], survey results on various clustering methods are presented. Nayyar et al. [17] and Abbasi et al. [18] report that the energy efficiency is one of the major design issues in wireless sensor networks. In References [19,20,21], low-energy adaptive clustering hierarchy (LEACH) and its extended versions are presented. LEACH is one of the first protocols proposed for clustering. In LEACH, each node is independent in terms of making the decision to become a cluster head, but this does not guarantee the creation of a uniform cluster head distribution across the network. In Reference [22], an efficient clustering method, which can be used for the network of a large number of nodes, is presented. In Reference [16], hybrid energy-efficient distributed (HEED) clustering is presented. The HEED guarantees the creation of a fairly uniform cluster head distribution across the network. As mentioned above, clustering is an effective topology control approach, but most of the clustering methods are designed to prolong the lifetime of a wireless sensor network.

In our previous work [23], we proposed a distributed self-organized cluster-based fusion tree generation (FTG) algorithm. The FTG takes into account the fusion workload of nodes instead of the residual energy of nodes, when it selects cluster heads. In Reference [23], we showed that the performance of the FTG is better than the performance of two naive methods. However, we found that the performance of the FTG is degraded in the case of a non-uniform distribution of targets. The FTG models the fusion workload of a radar by counting the number of its neighbor nodes. This model assumes that targets are uniformly distributed. We can infer that this model induces the performance degradation of FTG in the non-uniform distribution of targets.

In this paper, we present a non-uniform FTG (NU-FTG) algorithm which can generate a balanced fusion tree for the two-tier hierarchical fusion process on the DMRS. The NU-FTG is based on clustering methods. It elects a set of cluster heads whose role is the same as that of the local fusion node, and the elected cluster heads can cover the other radars via one hop. The execution of NU-FTG is fully distributed. All nodes execute NU-FTG independently with the information of their neighbors. Any prior information, such as the appropriate number of local fusion nodes, predefined tree structure, or position of radars, is not required. We evaluate the performance of the NU-FTG in the OPNET (Optimized Network Engineering Tool), network simulator. Every fusion cycle, the number of processed tracks in the SIAP is collected, and the average number of the collected tracks is calculated. The simulation results show that the NU-FTG performs better than FTG [23] and HEED [16], not only in uniform, but also in non-uniform distribution of targets. Additionally, the simulation results show that the performance of the NU-FTG is not degraded, even though the start time of the radars are not synchronized.

The rest of this paper is organized as follows: Section 2 presents the fusion process model for the two-tier hierarchical fusion architecture to provide background information; Section 3 presents the problem statement and the methodology overview, defining the fusion tree generation problem, and provides the requirements of the problem; Section 4 presents an algorithm overview and detailed descriptions of the NU-FTG; Section 5 shows the evaluation results and discussions. It provides detailed experimental configurations, and the results and discussions on three different configurations; finally, Section 6 presents conclusions and future works.

## 2. Preliminaries: The Fusion Process Model

### 2.1. Two-Tier Hierarchical Fusion Process

**Figure 1:**
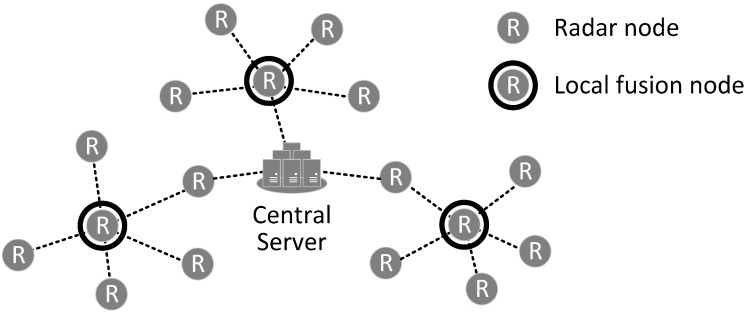
Two-tier hierarchical fusion process model.

Figure 1 describes the two-tier hierarchical fusion system which consists of vehicle-mounted radars, local fusion nodes that are elected among radars, and a central server. Radars are divided into distinct clusters. For each cluster, one of the radars becomes a cluster head whose role is like that of a local fusion node, and the others become children of their local fusion node. In a cluster, child radars should be connected to their local fusion node via one hop. On the other hand, local fusion nodes can be connected to the central server via multiple hops. Radars form a mobile ad-hoc network to support the multi-hop connection between the local fusion nodes and the central server. This structure can be represented via a tree structure called a fusion tree. The fusion tree is defined as follows: radars are leaf nodes; local fusion nodes are internodes; and the central server is the root of the fusion tree.

The capabilities of radars:

- Radars are equipped with an anti-air radar sensor whose detection range is a circle with a radius of *d* km;
- Radars have a tracker which creates local tracks with a series of measurements from the anti-air radar sensor; and
- Any radar can be elected as a local fusion node which executes local fusion processes to create the local SIAP.

The capabilities of the central server:

- The central server executes the global fusion process to create global SIAP with the tracks from local fusion nodes; and
- The central server is equipped with enough computing power to process all received tracks in a fusion cycle.

The two-tier hierarchical fusion process consists of tracking, local fusion, and global fusion processes. These processes are arranged in a hierarchical structure in the fusion tree. Radars, including local fusion nodes, execute the tracking process to create local tracks with their measurements. Local fusion nodes execute the local fusion process to create the local SIAP with the local tracks from their child radars and their own tracker. The central server executes the global fusion process to create the global SIAP with tracks from the local fusion nodes.

### 2.2. The Local Fusion Process Model

This section presents the time-delay model of the local fusion process at the local fusion nodes. The time-delay model of global fusion process is not included, since it is assumed that the central server is equipped with enough computing power to process all received tracks in a fusion cycle. The time-delay model is used in the NU-FTG and local fusion processes. In the NU-FTG, this model is used to calculate the score of each radar. The NU-FTG uses the calculated score of the radars to elect local fusion nodes. In the local fusion process, this model is used to simulate the time-delay of the fusion process at the local fusion nodes.

Tracks are the estimated states of targets, and targets are real objects. The state of a track is estimated from a set of measurements that have originated from the same target. The state variable of a track K is:

(1) $$\hat{K}=\left[X,Y,Z,V_{x},V_{y},V_{z}\right].$$

In Equation (1), $\hat{K}$ is the state estimation of a track K, [X,Y,Z] is the estimated position, and $\left[V_{x},V_{y},V_{z}\right]$ is the estimated velocity.

**Figure 2:**
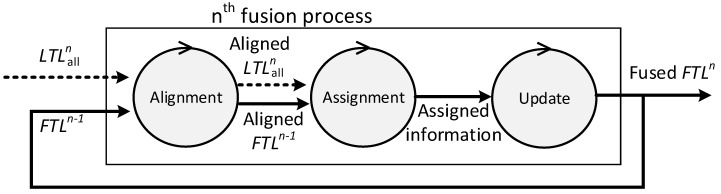
Fusion process model at local fusion nodes.

Figure 2 describes the fusion process model of the local fusion process. The fusion process consists of alignment, assignment, and update. Every fusion cycle, local fusion nodes start the fusion process to create the local SIAP with the tracks from their child radars and their own tracker. The inputs of the *n*th fusion process of a local fusion node which has N child radars is as follows:

- $LTL_{all}^{n}=\text{\{}LTL_{i}^{n}\;|\;i=1,\;2,\;\ldots ,\;N\}$, is a set of local track lists. The $LTL_{i}^{n}$, which stands for local-track-list, is a set of local tracks received from radar i after the (*n* − 1)th fusion process.
- $FTL^{n-1}$, which stands for fused-track-list, is a set of tracks in the local SIAP created at the (*n* − 1)th fusion process. It is empty for the first fusion process.

In the alignment, the states of tracks are extrapolated to the current time before using tracks that are created or updated at different times. It is assumed that tracks make the uniform motion of a straight line. The execution time of the alignment process can be modeled as:

(2) $$T_{alg}=\left(\left|FTL^{n-1}\right|+\sum_{i=1}^{N}\left|LTL_{i}^{n}\right|\right)\times C_{1}.$$

In Equation (2), $C_{1}$ is the execution time of the alignment process for a track, and $\left|FTL^{n-1}\right|$ and $\left|LTL_{i}^{n}\right|$ stand for the number of tracks in the fused-track-list and the local-track-list, respectively.

In the assignment, local tracks are assigned to the tracks in the local SIAP. A local track is assigned to the one of the tracks, originating from the same target, in the local SIAP. The association cost, which is based on the distance between two tracks, is used to decide whether two tracks originated from the same target or not. The following procedures are repeated for every $LTL_{i}^{n}$, where i=1, 2, …, N:

- Association costs of every pair of tracks (a,b) where $a\in LTL_{i}^{n}\;\mathrm{and}\;\;b\in FTL^{n-1}$ are calculated.
- Based on the association costs and assignment rules, some of the tracks in $LTL_{i}^{n}$ are assigned to tracks in $FTL^{n-1}$. Every track of $LTL_{i}^{n}$ is assigned by, at most, one of the tracks in $FTL^{n-1}$, and vice versa.

The execution time of the assignment can be modeled as:

(3) $$T_{asg}\approx \sum_{i=1}^{N}\left(\left|LTL_{i}^{n}\right|\times (\left|FTL^{n-1}\right|\times C_{2}+C_{3}\right)).$$

In Equation (3), $C_{2}$ is the execution time of the process to obtain the association cost of two tracks, and $C_{3}$ is the execution time of the process to assign a track in $LTL_{i}^{n}$ to a track in $FTL^{n-1}$.

In the update, the tracks in the local SIAP are updated with the assigned local tracks; unassigned tracks in the local SIAP are removed, and unassigned local tracks are added to the local SIAP. After this process, $FTL^{n-1}$ becomes $FTL^{n}$. The execution time of the update can be modeled as:

(4) $$T_{upt}\approx \left|FTL^{n-1}\right|\times C_{4}.$$

In Equation (4), $C_{4}$ is the execution time of the process to update a track in $FTL^{n-1}$. 

The constant parameters $C_{1},\;C_{2},\;C_{3}$, and $C_{4}$ are dependent on the computing power of a local fusion node. Thus, we implemented and ran the fusion process on our target hardware which is configured as follows: OS (QNX 6.3), CPU (Pentium M processor @ 1.6 GHz), and RAM (2 GB). In this configuration, $C_{1}$ is 356,972 nanoseconds; $C_{2}$ is 74,800 nanoseconds; $C_{3}$ is 79,398 nanoseconds; and $C_{4}$ is 33,364 nanoseconds.

## 3. Problem Statement and Methodology

### 3.1. The Fusion Tree Generation Problem

The computing power of the local fusion nodes and the network bandwidth are limited. Therefore, the fusion tree should balance local tracks across local fusion nodes to increase the number of processed tracks in a fusion cycle.

Problem overview:

- Electing a set of local fusion nodes that can cover the other radars via one hop;
- Electing an appropriate number of local fusion nodes by considering the number of tracks; and
- Electing more local fusion nodes in the area where there are more tracks.

Requirements:

- No predefined tree structure, such as the number of local fusion nodes and child radars, is used;
- All radars should be connected to one of the local fusion nodes in one hop, or become local fusion nodes; and
- Fully-distributed, radars need to know the information of their neighbors, only.

### 3.2. Methodology Overview

The purpose of this research work is not developing the fusion process itself, but designing an algorithm for fusion tree generation. We propose a non-uniform FTG (NU-FTG) algorithm to generate the fusion tree for the two-tier fusion process. In the NU-FTG, the score of a radar is in proportion to the number of local tracks of the radar and its neighbors. Radars with higher scores get more chances to become local fusion nodes.

Numerous nodes are required to see the impact of the different fusion trees on the performance of the fusion process. Therefore, the NU-FTG is evaluated in the OPNET (Optimized Network Engineering Tool), network simulator. The NU-FTG algorithm, and the fusion process, are implemented on the node model of the OPNET. In the case of NU-FTG, all operations of the algorithm are implemented on the process model of the *traffic_engine* application. On the other hand, the fusion process is implemented as a time-delay model. The time-delay model is the sum of $T_{alg}$, $T_{asg}$, and $T_{upt}$ defined in Equations (2)–(4), respectively.

The performance, defined as the number of process tracks in a fusion cycle, of the NU-FTG was compared to the performance of the FTG [23], and the HEED [16]. The HEED is a clustering method for wireless sensor networks. The HEED is able to generate a fusion tree which fulfills the requirements that are presented in Section 3.1. However, the HEED uses residual energy which is not available in the dynamic multi-radar system. In this paper, we assume that the residual energy of every node is the same.

## 4. NU-FTG Algorithm

### 4.1. Algorithm Overview

We propose using a clustering method to generate a fusion tree for the two-tier hierarchical fusion process. Clustering sensor nodes is an effective topology control approach [16]. Research on wireless sensor networks (WSN), such as References [16,17,18,19,20,21,22], have applied the clustering method to prolong the network lifetime by reducing the energy consumption on sensor nodes. On the other hand, the focus of this research is to increase the number of processed tracks of the two-tier fusion process. Therefore, the existing clustering method is not suitable for generating the fusion tree of the two-tier fusion process.

We have developed a non-uniform FTG (NU-FTG), a novel distributed fusion tree generation algorithm for the dynamic multi-radar system (DMRS). The NU-FTG elects a set of cluster heads which can cover the other radars in one hop. Some of the radars, elected as cluster heads, become local fusion nodes. The other radars become members of one of the local fusion nodes in one hop. Every radar runs the algorithm with the node information, defined in Table 1, of its neighbors. Other information, such as the position of radars, predefined tree structure, and appropriate number of cluster heads, is not required.

The NU-FTG uses three parameters to elect local fusion nodes and balance local tracks across local fusion nodes. The three parameters, *nscore*, *fscore*, and *pdt_ftime*, are defined as follows:

*nscore* = *OT + NT + Nbr*. (5)

In Equation (5), the *nscore* reflects the number of own tracks, neighbors’ tracks, and the number of neighbors.

*fscore* = *OT + CT + Chd*. (6)

In Equation (6), the fscore reflects the number of own tracks, child radars’ tracks, and the number of child radars.

*pdt_ftime* = *PdtModel(OT + CT)*. (7)

In Equation (7), the *PdtModel* predicts the time-delay of a local fusion process. A local fusion node can calculate $T_{alg}+T_{asg}+T_{upt}$ with the *PdtModel.* The number of local tracks and fused tracks in the local SIAP are required to calculate $T_{alg}+T_{asg}+T_{upt}$. The number of local tracks is given as *OT + CT*. However, the number of fused tracks in the local SIAP is not available before starting the fusion process. In the *PdtModel*, it is assumed that the number of fused tracks is same to the *OT + CT* by considering the worst case. The *pdt_ftime* is used to decide whether a local fusion node is available or not. A local fusion node is available only when the *pdt_ftime* is smaller than a fusion cycle.

The NU-FTG algorithm consists of two parts: role selection and member migration. In the first part, the *nscore* and *pdt_ftime* are used to select the role of radars. Some radars that have available local fusion nodes in one hop distance become a member of the local fusion node with the highest *nscore*. Others, whose *nscore* is bigger than their neighbors’, are elected as local fusion nodes. The others wait for a specified time duration and retry to select their role. As a result, radars are elected as a local fusion node only when they have no other available local fusion nodes in one hop. With this approach, the NU-FTG not only places more local fusion nodes to the area where more tracks are, but also prevents electing surplus local fusion nodes. In the second part, the *fscore* is used to balance the fusion workload among local fusion nodes. FM nodes migrate to the one of the neighbor local fusion nodes which has the lowest *fscore*.

### 4.2. Algorithm Descriptions

Algorithm 1 describes the pseudocode of the NU-FTG, which consists of two different parts. Before the start of the algorithm, radars initialize their variables: Sts, myCh, $S_{rs}$, $S_{ch}$, and $S_{fch}$. The Sts, which stands for status of a node, is initialized as RS (role selection). myCh, used to save the ID of the cluster head of a node, is initialized as “none”. $S_{rs}$, which stands for the set of RS nodes in one hop, is initialized as ∅. $S_{ch}$, which stands for the set of available TCH or FCH nodes in one hop, is initialized as ∅. $S_{fch}$, which stands for the set of available FCH nodes in one hop, is initialized as ∅.

In the first part of the NU-FTG, radars repeat the loop until they have decided their status as FCH or FM. At the beginning of the loop, $S_{rs}$ is updated with node-information of RS nodes in one hop, and $S_{ch}$ is updated with the node-information of available TCH or FCH nodes in one hop. Initially, the status of nodes is RS, and RS nodes become TM or TCH nodes. TM and TCH nodes become FM and FCH nodes, respectively; or both TM and TCH nodes change back to RS. RS nodes select their role with node-information of their neighbors. Some RS nodes that have available TCH or FCH nodes in their $S_{ch}$ change their status to TM. Others, whose *nscore* is greater than or equal to the *nscore* of nodes in $S_{rs}$, change their status to TCH. The others stay in RS. TM nodes decide to become FM nodes or not. A TM node selects the TCH or FCH node which has the highest *nscore* in $S_{ch}$. as its parent; and the TM node sends a *req_join* message to the selected parent. If the TM node is allowed to join, it updates its myCh with the ID of the selected parent and it becomes a FM node. Otherwise the TM node changes its status to RS. TCH nodes decide to become FCH nodes or not. A TCH node waits for the specified time duration which can be decided by considering the network latency. If the TCH node receives any *req_join* message or has no available TCH or FCH nodes in $S_{ch}$, then it changes its status to FCH. Otherwise it changes its status to RS.

In the second part of the NU-FTG, FM nodes repeat the loop to balance tracks across the local fusion nodes. At the beginning of the loop, $S_{fch}$ is updated with the node-information of available FCH nodes in one hop. A FM node selects the FCH node, which has the lowest *fscore*, in $S_{fch}$. The FM node can migrate to the selected FCH node only when the *fscore* of the selected FCH node is less than the *fscore* of its current FCH node. Additionally, a FM node migrates to the selected FCH node with the specified probability of ϵ. This condition is required to prevent nearby FM nodes from continuing to change their FCH node. If the probability is too small or large, it takes a significant amount of time until the fusion tree is stabilized. The probability ϵ can be acquired practically with some experiments. If an FM node decides to migrate, then it sends *req_join* to the selected FCH node and wait for the specified time duration. If the FM node is allowed to join, it migrates to the selected FCH node.

**Algorithm 1.** Non-uniform Fusion Tree Generation (NU-FTG) algorithm1:▷ Initialization26:▷ Second part—Member Migration2:Sts ← RS27:**while**(Sts = FM)3:myCh ← none28:   $S_{fch}$←{v:v is available FCH in 1-hop}4:$S_{rs}$ ← $S_{ch}$ ← $S_{fch}$ ← ∅29:   **if**(*fscore* of myCh−min *fscore* in $S_{ch}>$ my *fscore*)5:
30:  **if**(*rnd_uniform*(1)≤ϵ) ▷ *rnd_uniform*(1) = 0~16:▷ First part—Role selection31:    tmpCh ← *Id* of min *fscore* node in $S_{ch}$7:**while**(Sts ≠ FCH and Sts ≠ FM)32:    Send req_join to tmpCh8:   $S_{rs}$←{v:v is RS node in 1-hop}33:  **if**(allowed)9:   $S_{ch}$←{v:v is available TCH or FCH node in 1-hop}34:    Send *dis_join* to myCh10:   ▷ RS nodes35:    myCh ← tmpCh11:   **if**(Sts = RS)

12:  **if**($S_{ch}$
≠
∅) Sts ← TM

13:  **else if**(my *nscore*≥ max *nscore* in $S_{rs}$) Sts←TCH

14:   ▷ TM nodes

15:   **else if**(Sts = TM)

16:  Send *req_join* to the max *nscore* node in $S_{ch}$

17:  **if**(allowed)

18:   myCh ← *Id* of max *nscore* node in $S_{ch}$

19:   Sts ← FM

20:  **else** Sts ← RS

21:   ▷ TCH nodes

22:   **else if**(Sts = TCH)

23:  Wait for *req_join* from neighbors

24:  **if**(received any *req_join* or $S_{ch}=\emptyset$) Sts←FCH

25:  **else** Sts ← RS



## 5. Evaluation and Discussion

### 5.1. Experiment Configuration

**Figure 3:**
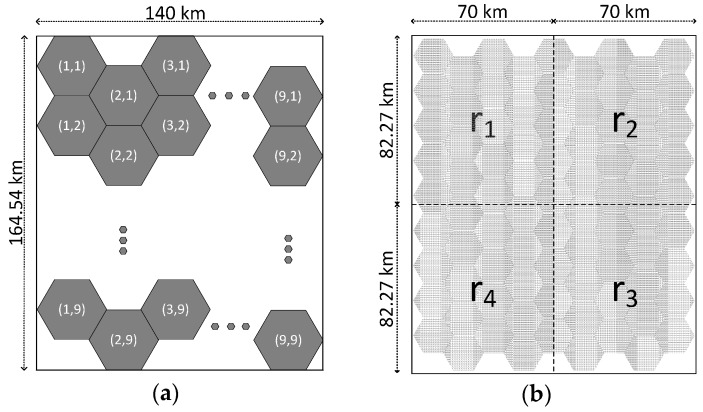
The definition of the surveillance area for evaluation: (**a**) surveillance area consisting of 9 × 9 cells; and (**b**) surveillance area is divided into four regions.

For all of experiments, 81 radars are distributed in the surveillance area, which consists of 9 × 9 cells as described in Figure 3a. A cell is a hexagon which is covered by a circle with a radius of 10 km. A radar is assigned at each cell, and a radar is randomly located within 2 km from the center of its assigned cell. The detection range of a radar is 15 km, and some of the detection range of a radar overlaps with the detection range of its neighbors.

We define the simulation environment with three parameters. The first parameter, $N_{tgt}$, is the number of deployed targets. The second parameter, ΔT, is the varied start time of radars. A radar starts the NU-FTG algorithm at a randomly-selected time between 0 and ΔT. The third parameter, $P_{r_{\#}}$, is the percentage that targets are deployed in the region $r_{\#}$. As shown in Figure 3b, the surveillance area is divided into four regions. Some of the targets are called regional targets which belong to one of the regions. Regional targets are uniformly distributed and move as random waypoint models in their region. The other targets are non-regional targets that do not belong to one of the regions. Non-regional targets are uniformly distributed and move as random waypoint models in the entire surveillance area.

### 5.2. Varied Number of Targets, Uniformly Distributed

In this section, we evaluate the performance of the HEED, FTG, and NU-FTG for a varied number of targets that are uniformly distributed. The parameters for the simulation are configured as follows:

- $N_{tgt}=200,\;400,\;\ldots ,\;or\;2000,$
- ∆T=0, and
- $P_{r_{1}}=P_{r_{2}}=P_{r_{3}}=P_{r_{4}}=0\%.$

We executed three experiments for HEED, FTG, and NU-FTG, respectively. An experiment consists of 10 sequences with the different number of targets: 200, 400,…, or 2000. Each sequence consists of 10 runs with the different random seeds: 1, 2,…, or 10. Each run continues for 300 s. Every second, the number of tracks in the global SIAP are collected. The result of a run with a random seed is $N_{AVG}^{seed}$, which stands for the average number of tracks in the global SIAP. Finally, the result of a sequence is the average of $N_{AVG}^{1\sim 10}$.

**Figure 4:**
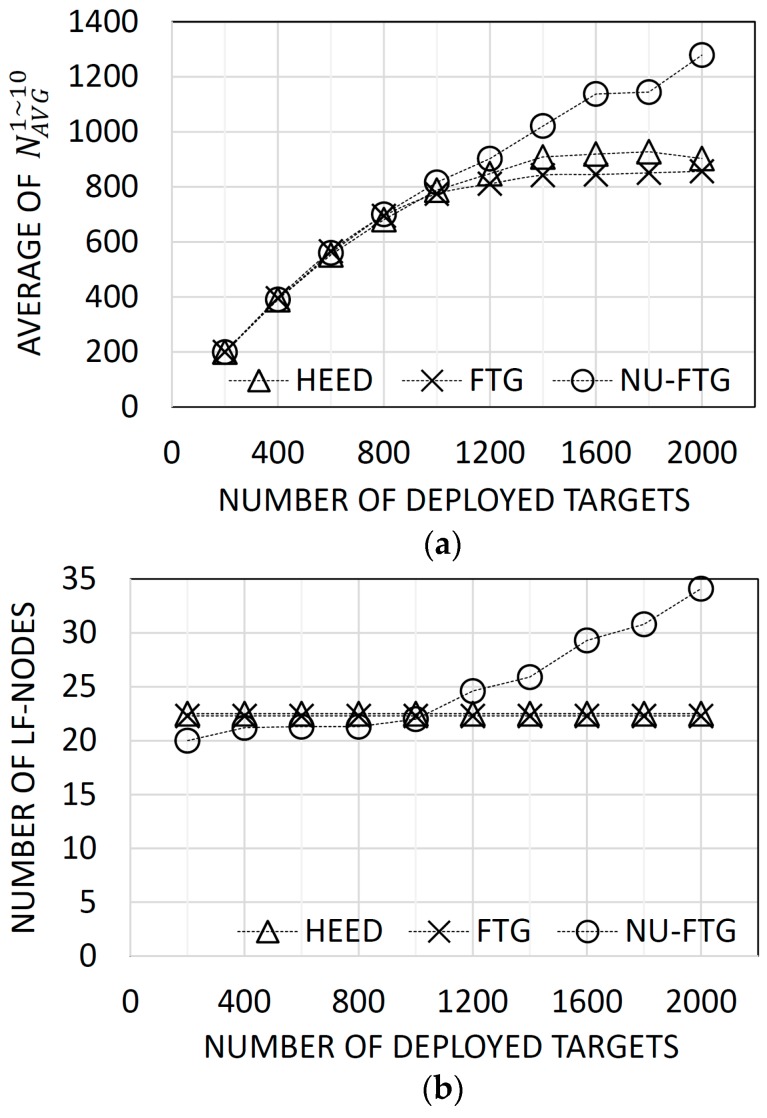
Results of experiments for varied number of uniformly distributed targets: (**a**) the average number of collected tracks for each sequence; and (**b**) the number of created local fusion nodes.

Figure 4a presents the average number of tracks in the global SIAP. The performance of the NU-FTG is similar to the performance of HEED and FTG when the number of deployed targets are less than 1200. However, the NU-FTG performs better than HEED and FTG when the number of deployed targets are 1200 or more.

The HEED was designed to distribute the traffic of nodes across cluster heads (local fusion nodes) by balancing their number of children. In these experiments, targets and radars are uniformly distributed, and the expected number of tracks of each radar is identical. As a result, the HEED can generate a balanced fusion tree. Thus, the performance of the HEED is similar to the performance of the FTG, and the NU-FTG when the number of deployed targets is less than 1200.

The performance of the HEED and FTG become worse than the performance of NU-FTG as the number of deployed targets increases. The performance degradation of the HEED and FTG might be induced by the fixed number of local fusion nodes. Figure 4b shows that the number of local fusion nodes of the HEED and FTG are fixed. Thus, the performance of a fusion process is limited by the fixed number of local fusion nodes when the HEED or FTG is applied. On the other hand, the number of local fusion nodes of the NU-FTG increases as the number of deployed targets increases.

### 5.3. Regional Targets, Non-Uniformly Distributed

In this section, we evaluate the performance of the HEED, FTG, and NU-FTG for varied $P_{r_{1}}$. The parameters for the simulation environment are configured as follows:

- $N_{tgt}=1200,$
- ΔT=0, and
- $P_{r_{1}}=10,\;30,\;50,\;70,\;or\;90\%$/$P_{r_{2}}=P_{r_{3}}=P_{r_{4}}=0\%$

We executed three experiments for HEED, FTG, and NU-FTG, respectively. An experiment consists of five sequences with different $P_{r_{1}}$: 10, 30,..., or 90%.

**Figure 5:**
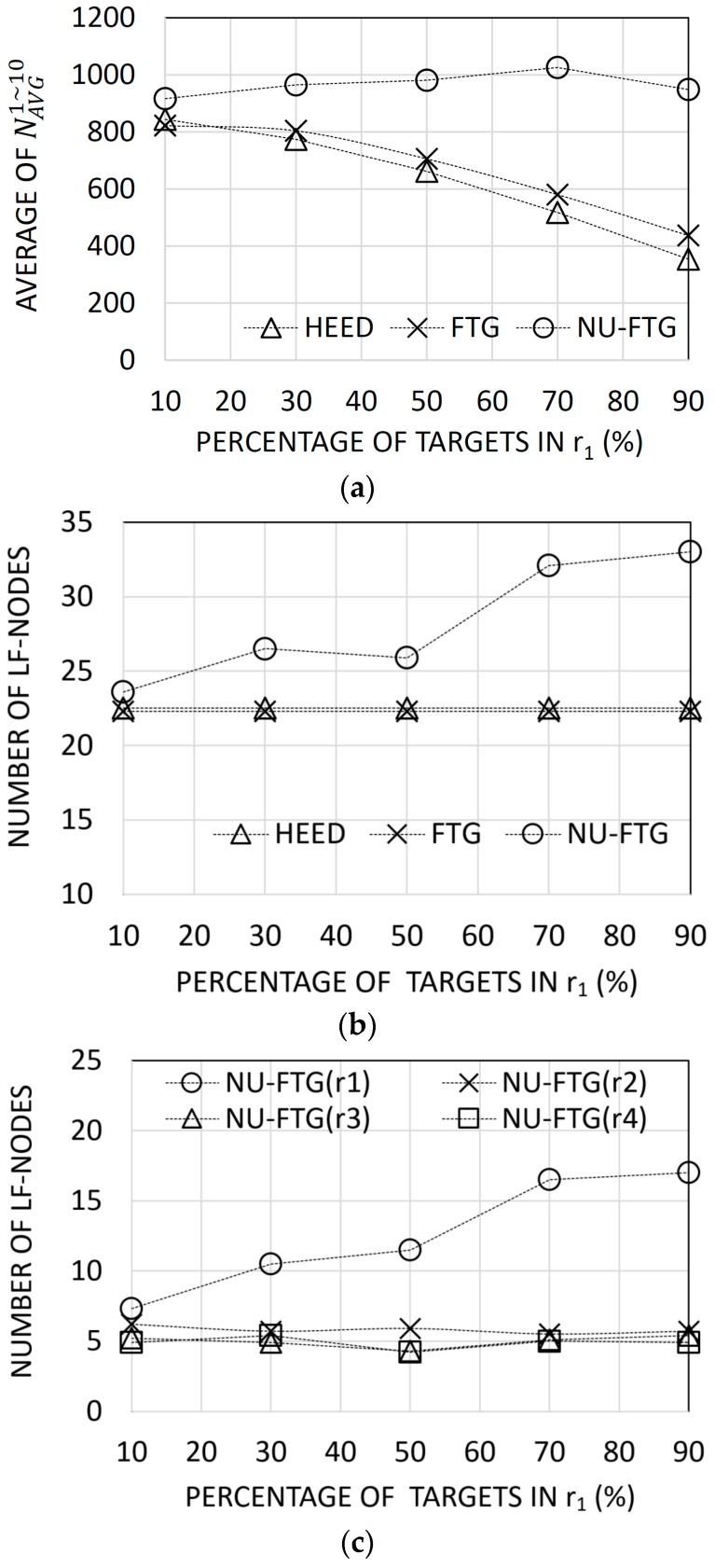
Results of experiments for non-uniformly distributed regional targets: (**a**) the average number of collected tracks for each sequence; (**b**) the number of created local fusion nodes for hybrid energy-efficient distributed (HEED), fusion tree generation (FTG), and non-uniform FTG (NU-FTG); and (**c**) the number of created local fusion nodes of each of region for NU-FTG.

Figure 5a shows that the NU-FTG performs better than HEED and FTG. The performance gap between NU-FTG and the others increases as the percentage of deployed targets in $r_{1}$ increases. This result can be explained with Figure 5b,c. The number of deployed targets in $r_{1}$ increases as the percentage of deployed targets in $r_{1}$ increases. However, in Figure 5b, the number of local fusion nodes increases only when the NU-FTG is applied. Especially, in Figure 5c, the number of local fusion nodes in $r_{1}$ increases. Therefore, the performance of the fusion process for the non-uniformly distributed targets is not degraded only when the NU-FTG is applied.

### 5.4. Unsynchronized Start Time

This section presents the performance of the NU-FTG for the varied Δ*T* in the uniform distribution of targets. The HEED and FTG do not support unsynchronized start times of radars; thus, we did not execute experiments for HEED and FTG. The parameters for the simulation environment are configured as follows:

- $N_{tgt}=1200,$
- ΔT=0, 10, 20, 30, 40, or 50, and
- $P_{r_{1}}=P_{r_{2}}=P_{r_{3}}=P_{r_{4}}=0\%$

**Figure 6:**
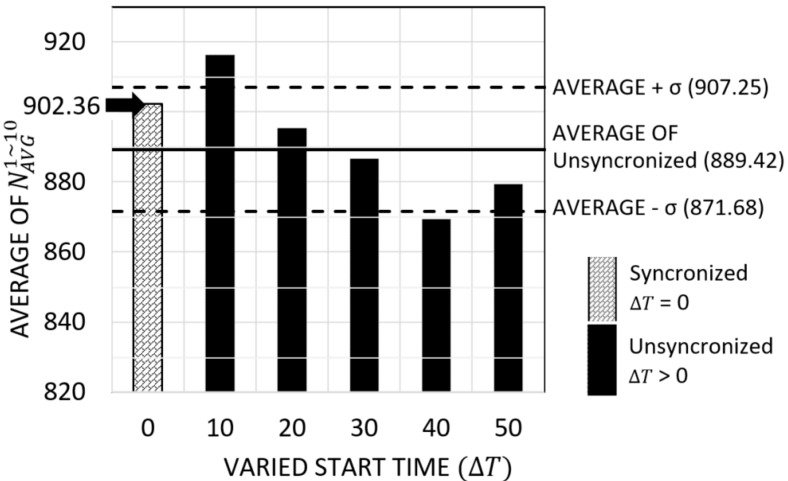
Results of experiments for an unsynchronized start time.

We executed an experiment for NU-FTG. The experiment consists of six sequences with different ΔT: 0, 10,…, or 50 s. A radar starts the NU-FTG at a randomly selected time between 0 and ΔT seconds. Figure 6 shows the results of this experiment. The first sequence, whose ΔT is 0, is the synchronized case. In the synchronized case, all nodes start the NU-FTG at around the same time. The other sequences are unsynchronized cases. The average number of collected tracks and the standard deviation of unsynchronized cases is calculated. As shown in Figure 6, the number of collected tracks of the synchronized case is 902.36, which is within one standard deviation from the average number of collected tracks of the unsynchronized cases. This result shows that the performance of NU-FTG in the unsynchronized cases are similar to the synchronized case, and we can infer that the NU-FTG can guarantee its performance, even though the start time of nodes are not synchronized.

## 6. Conclusions

The purpose of this research work is to increase the number of processed tracks of a two-tier hierarchical fusion process in a fusion cycle by creating a balanced fusion tree. The balanced fusion tree can balance fusion workload across local fusion nodes. Clustering sensor nodes is an effective topology control approach [16]; and it can be used to create fusion tree for the two-tier hierarchical fusion process. However, the normal clustering methods for wireless sensor networks do not guarantee to create a balanced fusion tree. In [12], we proposed the fusion tree generation (FTG) algorithm which considers fusion workload. However, the FTG models the fusion workload of a radar by counting the number of its neighbor nodes. Therefore, the FTG does not guarantee to create a balanced fusion tree when targets are non-uniformly distributed.

In this paper, we have proposed the non-uniform FTG (NU-FTG) algorithm executed in a fully distributed manner. In the NU-FTG, the fusion workload of a node is in proportion to the number of its own and its neighbors’ tracks. With this approach, the NU-FTG is guaranteed to create a balanced fusion tree not only when targets are uniformly distributed, but also when targets are non-uniformly distributed. The performance of the NU-FTG was compared to the performance of FTG [23] and HEED [16] in the OPNET (Optimized Network Engineering Tool), network simulator; the simulation results are presented in Section 5. In Section 5.2, the NU-FTG performs better than FTG and HEED as the number of deployed targets increases. This result shows that the NU-FTG is more robust than the others in varied number of targets. In Section 5.3, the NU-FTG performs better than FTG and HEED in the non-uniform distribution of targets. In Section 5.4, the performance of the NU-FTG is evaluated for the unsynchronized start time of radars. Section 5.4 shows that the NU-FTG can guarantee the performance of two-tier fusion process, even though the start time of radars are unsynchronized.

The future work is to design the algorithm which considers the dynamic environments: movement and failure of nodes, and movement of targets as a large group. Additionally, we will design a constraint programming model to solve FTG problems; this model can be used to verify the performance of FTG algorithms.

## Figures and Tables

**Table 1:** Node information.

Information	Descriptions
*Id*	Identification number of a node
*Sts*	Status of a node ○ RS (Role Selection) ○ TM (Temporal Member) ○ FM (Final Member) ○ TCH (Temporal Cluster Head) ○ FCH (Final Cluster Head)
*Nbr*	Number of neighbors in one hop
*Chd*	Number of child nodes of a cluster head
*OT*	Number of own tracks of a node
*NT*	Number of tracks from neighbor nodes
*CT*	Number of tracks from child nodes
